# The Ovariectomized Rat as a Model for Studying Alveolar Bone Loss in Postmenopausal Women

**DOI:** 10.1155/2015/635023

**Published:** 2015-04-28

**Authors:** Bryan D. Johnston, Wendy E. Ward

**Affiliations:** ^1^Faculty of Applied Health Sciences, Brock University, St. Catharines, ON, Canada L2S 3A1; ^2^Centre for Bone and Muscle Health, Brock University, St. Catharines, ON, Canada L2S 3A1

## Abstract

In postmenopausal women, reduced bone mineral density at the hip and spine is associated with an increased risk of tooth loss, possibly due to a loss of alveolar bone. In turn, having fewer natural teeth may lead to compromised food choices resulting in a poor diet that can contribute to chronic disease risk. The tight link between alveolar bone preservation, tooth retention, better nutritional status, and reduced risk of developing a chronic disease begins with the mitigation of postmenopausal bone loss. The ovariectomized rat, a widely used preclinical model for studying postmenopausal bone loss that mimics deterioration of bone tissue in the hip and spine, can also be used to study mineral and structural changes in alveolar bone to develop drug and/or dietary strategies aimed at tooth retention. This review discusses key findings from studies investigating mandible health and alveolar bone in the ovariectomized rat model. Considerations to maximize the benefits of this model are also included. These include the measurement techniques used, the age at ovariectomy, the duration that a rat is studied after ovariectomy and habitual diet consumed.

## 1. Introduction

**Figure 1:**
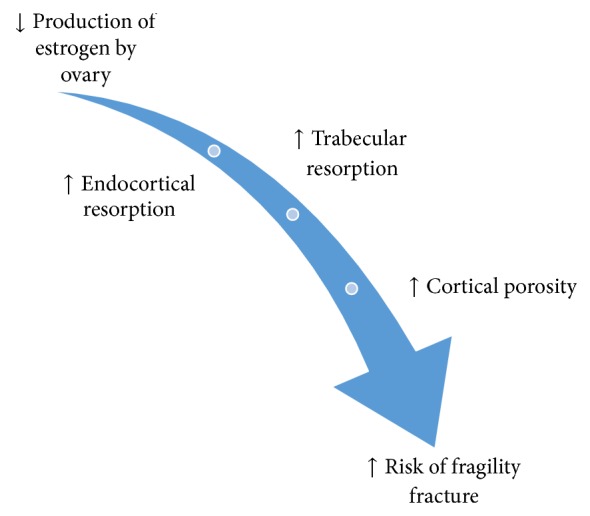
Overview of how osteoporosis develops after menopause. With the loss of endogenous estrogen production by the ovaries there is an increase in trabecular bone resorption, endocortical bone resorption, and cortical porosity that elevate a woman's risk of experiencing a fragility fracture.

A decline in ovarian production of estrogens at menopause often results in a rapid loss of trabecular microarchitecture, increased endocortical bone resorption, and increased cortical porosity; all culminating in the development of osteoporosis and the associated increased risk for fragility fracture (Figure 1) [1]. Specifically, the number of osteoclasts increases to a point where the rate of bone resorption exceeds the rate of bone formation [2].

Based on data from the WHO Global Burden of Disease project in 2000, an estimated 56 million people around the world experience disability caused by a fracture [3]. In 2010, there were 2.32 million new hip fractures in adults over 50 years of age worldwide, and approximately half of those hip fractures were due to osteoporosis in the femur neck (hip) [4]. Based on osteoporosis prevalence rates reported in the 2010 Census and NHANES (2005–2010), it is estimated that 10.2 million Americans over the age of 50 have osteoporosis while an additional 43.4 million have low bone mass that predisposes them to the development of osteoporosis [5]. It is well documented that women are disproportionally affected by osteoporosis compared to men, primarily due to the more sudden decline in estrogen production experienced at menopause whereas sex steroid levels decline more gradually in men [3–6]. In the majority of postmenopausal women, the risk of experiencing a fragility fracture exceeds the risk of developing invasive breast cancer, stroke, and cardiovascular disease combined [7]. Moreover, there is substantive morbidity [8] associated with a fragility fracture and an increased risk of death, especially within the first year after fracture [9].

## 2. Osteoporosis, Estrogen, and Tooth Loss

Osteoporosis not only increases a woman's risk of fragility fracture at the hip, spine, and wrist, but it is also associated with the loss of teeth and tooth supporting alveolar bone [10–13]. For example, osteoporosis at the lumbar vertebrae, femoral neck, or total hip is a significant predictor of molar tooth loss [10]. A 5-year longitudinal study of 404 postmenopausal women confirmed that women in the highest tertile of annual BMD loss at the lumbar spine and femoral neck had an adjusted relative risk of 1.38 and 1.27 for tooth loss, respectively [11]. In a longitudinal study of even greater duration, 7 years, the relative risk of tooth loss in 180 postmenopausal women (serum estradiol (E2) < 25 pg/mL) was 4.38 with each 1% annual decrease in whole body BMD [12]. An increased loss of alveolar bone height and decreased alveolar crestal and subcrestal bone mineral density, all critical for providing support for teeth, were also reported in a 2-year longitudinal study of 38 women with osteopenia and osteoporosis at the lumbar spine. Between the first and second molars in particular, estrogen-deficient (mean serum E2 < 30 pg/mL) women lost more alveolar crestal bone density compared to estrogen-sufficient (mean serum E2 > 40 pg/mL) women [13]. Because tooth retention [14, 15] and functional dentition [16, 17] are key determinants of nutritional status the maintenance of alveolar bone is important for overall health. Risk of many chronic diseases such as obesity, type 2 diabetes, cardiovascular disease, and some cancers is elevated by poor diet. Thus, strategies that preserve the skeleton at key sites of fragility fracture—hip, spine, and wrist—as well as alveolar bone in the jaw are important for healthy aging.

While estrogen replacement therapy (ERT) has been consistently shown to reduce fragility fractures at the hip, spine, and wrist [18, 19] the effect on tooth retention and preserving alveolar bone has been less studied. However, a study of 42,171 postmenopausal women (aged ≤ 69 years) over a 2-year period as part of the Nurses' Health Study cohort reported that current use of hormone replacement therapy (HRT: estrogen alone, in combination with progestin, or progestin alone) was associated with a 24% decrease in the risk of tooth loss. In women using conjugated estrogen alone, at a dose of 0.3 mg per day, the risk of tooth loss was 31% lower compared to nonusers [20]. In a cohort of 3,921 older women, median age of 81 years, current ERT (with or without progesterone) was associated with a 27% lower risk of tooth loss [21]. Another study showed that a group of postmenopausal women (72–95 years of age) who used ERT (reported as any use of estrogen) for greater than 8 years retained an average of 3.6 more teeth than women who never used ERT [22]. The duration of ERT (estrogen alone or in combination with progestogen) was also a significant predictor of total and posterior teeth remaining in a group of 330 postmenopausal Japanese women [23]. The mechanism behind tooth retention and ERT remains unclear, but one 3-year longitudinal study of 135 women aged 41–70 years concluded that women receiving 0.625 mg conjugated equine estrogen with or without 2.5 mg medroxyprogesterone acetate experienced a 0.9% increase in alveolar bone mass, as assessed by digitized radiographs, compared to nonusers [24]. HRT or ERT may therefore work to increase the stability of the tooth-supporting alveolar bone and thereby promote tooth retention and the opportunity to consume a wide variety of foods.

## 3. Tooth Loss and Nutritional Status

Retention of natural teeth is associated with healthier nutrient intakes that may have a role in prevention of chronic disease. For example, dietary calcium has been studied in relation to tooth loss because achieving recommended intakes of dietary calcium, in particular, is important for attenuating bone loss after menopause and during aging. As such, the recommended intake of calcium is 1200 mg per day in women aged 51–70 years and men over the age of 70 [25]. Among older men and women (≥65 years of age) with unknown smoking status, higher daily intake of calcium (884 versus 805 mg calcium) was associated with a greater number of teeth (≥21 versus 11–20 teeth) [14]. Another study reported lower tooth loss in a placebo-controlled 2-year study of nonsmoking women taking a calcium supplement of 500 mg per day; smoking women were excluded because smoking is a risk factor for tooth loss [12].

Fruit and vegetable intake in relation to tooth loss has also been studied. Data from NHANES III, a large cross-sectional study of Americans over the age of 50, showed a reduced number of posterior occlusal teeth associated with a lower daily intake of the recommended amount of fruit servings as reflected in a lower Healthy Eating Index (HEI) score and a higher BMI [16]. Additionally, having no posterior occlusal teeth was associated with a lower daily intake of the recommended amount of vegetable servings, also reflected in a lower HEI score [16]. Even when controlling for socioeconomic status, inadequate dentition (defined by <21 teeth remaining) was associated with reduced intakes of fruit (stone fruits and grapes/berries) and vegetable (stir-fried or mixed vegetables, sweetcorn/corn on the cob, mushrooms, lettuce, and soy beans/tofu) in a sample of 530 dentate Australian men and women over the age of 55 [17]. The link between tooth loss and reduced fruit and vegetable intake is important since a recent comprehensive review concluded that fruit and vegetable intake was associated with a reduced risk of chronic diseases such as hypertension, coronary heart disease, and stroke [26]. Interestingly the relationship between a reduced BMD in postmenopausal osteoporosis, higher rates of tooth loss, and reduced fruit and vegetable intake comes full circle given the study of 670 postmenopausal Chinese women that found higher fruit and vegetable intake was associated with higher whole body, lumbar spine, and hip BMD. Specifically, a daily increase of 100 g of fruits and vegetables was associated with a 6, 10, and 6 mg/cm^2^ higher BMD at the whole body, lumbar spine, and hip, respectively. [27].

**Figure 2:**
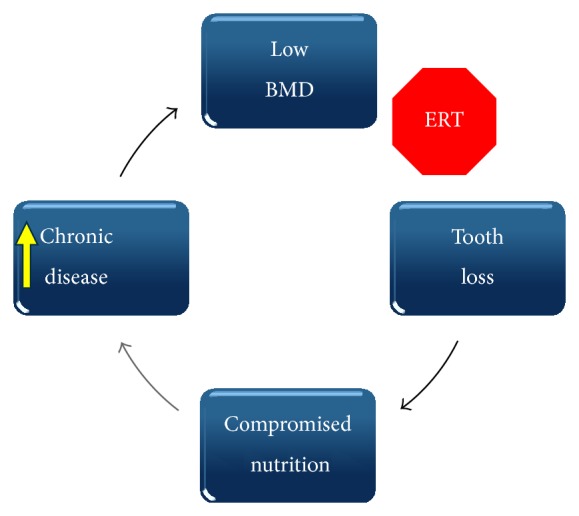
Cyclical relationship among low BMD, tooth loss, compromised nutrition, and risk for chronic disease. Estrogen or hormone replacement therapy (ERT, HRT) or other interventions that benefit other skeletal sites (hip, spine, and wrist) may prevent or slow the progression from low BMD to tooth loss. Retention of natural teeth allows individuals to eat a more healthful diet associated with a reduced risk of developing a chronic disease.

The relationship between osteoporosis, tooth loss, and compromised nutrition may prove to be cyclical (Figure 2) since compromised nutrition could exacerbate osteoporosis. As discussed in Section 2, HRT or ERT has been shown to promote tooth retention and may intervene in this self-perpetuating, negative cycle of tooth loss and the consequent higher risk of chronic disease development. Moreover, there are other pharmacological agents or diet interventions that may prove useful in stopping the cycle shown in Figure 2. The ovariectomized rat can be used to evaluate the effectiveness of an intervention for preserving alveolar bone. Findings from these studies provide an important step in developing interventions to promote and support bone health, including the retention of natural teeth, for postmenopausal women.

## 4. The Ovariectomized Rat Model

The ovariectomized (OVX) rat model is the approved preclinical model by the Food and Drug Administration (FDA) [28] for studying how the decline in endogenous estrogen production by the ovaries at menopause leads to postmenopausal osteoporosis and how potential interventions can preserve bone metabolism in this state. Although the FDA guidelines do not specify which strain of rat to use, it is important to be aware that there can be differences in bone mineral density, bone size, and biomechanical bone strength among inbred rat strains [29]. Interventions include pharmacological agents as well as lifestyle strategies such as diet. By 12 weeks of age, the female Sprague-Dawley rat, among the most common strains studied, has reached sexual maturity and has achieved peak bone mass for the whole body, femora, and tibiae [30]. Peak bone mass was defined as the point at which the rat skeleton had accrued its highest amount of areal BMD determined by dual-energy X-ray absorptiometry (DXA) in the whole body, femur, and tibia. However, longitudinal bone growth continues in the female rat until the epiphyseal growth plates close. At 12 weeks of age, the distal tibia growth plate has closed while the proximal tibia and lumbar vertebral growth plates remain open until 15 and 21 months, respectively [31]. Despite the continued skeletal growth, rats are commonly ovariectomized at 12 weeks of age since the rats are sexually mature and therefore capable of modeling bone loss due to estrogen deficiency [32]. By 9 months of age, longitudinal bone growth at the proximal tibia metaphysis has slowed to 3 *μ*m/day, while growth at the lumbar vertebral body has slowed to <1 *μ*m/day [33].

Two developmental stages have been used to describe the adult rat skeleton: “mature” from 3–6 months of age and “aged” ≥ 6 months of age. Skeletal growth is rapid from 1–3 months, reduced from 3–6 months, and negligible past 6 months of age [34]. In addition to continued longitudinal bone growth, the extent of ovariectomy-induced bone loss is dependent on both the skeletal site and the time since ovariectomy. For example, in the proximal tibia, a significant decrease in trabecular bone volume is observed 2 weeks after ovariectomy compared to sham control with a plateau by 14 weeks after ovariectomy [35]. At the femoral neck, a significant decrease in trabecular bone volume occurred at 4 weeks after ovariectomy with a plateau by 39 weeks after ovariectomy [36]. The lumbar vertebrae were much more resistant to ovariectomy-induced changes in trabecular bone volume than either the proximal tibia or femoral neck. It was not until 7 weeks after ovariectomy that a decrease in trabecular bone volume was significant and reached a plateau between 39 and 77 weeks after ovariectomy [37].

**Figure 3:**
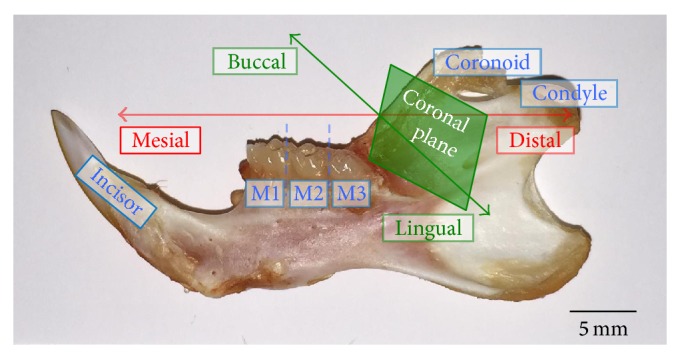
Hemimandible from a 6-month-old Sprague-Dawley rat. From left to right: incisor, 1st molar (M1), 2nd molar (M2), 3rd molar (M3), the coronoid process, and condyle. Mesial is the front, distal the back, buccal the lateral side, lingual the medial side, and the coronal plane divides the mandible into mesial and distal halves (photo by B. Johnston).

**Figure 4:**
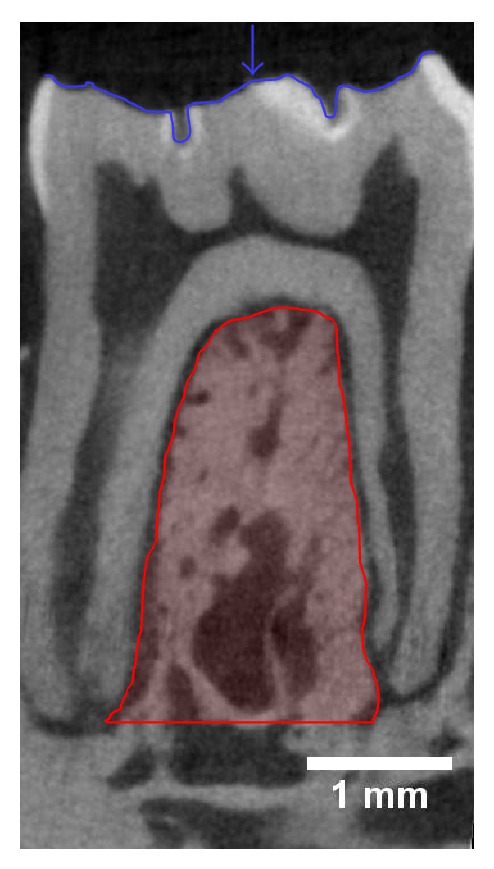
*μ*-CT 2D sagittal slice through molar 1 (M1) of a 6-month-old Sprague-Dawley rat with interradicular septum. The interradicular septum is highlighted in red and extends from the furcation roof to the root apices. The occlusal surface of M1 is also highlighted in blue and the arrowhead denotes the approximate area of the central sulcus. The left side of the image is mesial and the right side is distal (image by B. Johnston).

**Figure 5:**
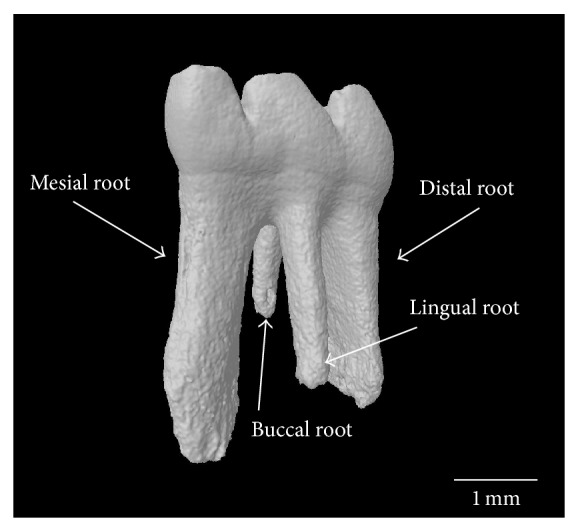
*μ*-CT 3D reconstructed image of the four roots of molar 1 (M1). The mesial, distal, buccal, and lingual roots enclose the alveolar bone of the interradicular septum (image by B. Johnston).

**Figure 6:**
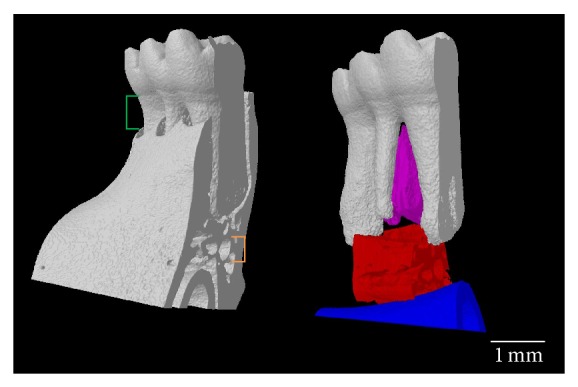
Landmarks to define ROI of alveolar bone at molar 1 (M1). M1 in its bony socket (left) with the distance between the cementoenamel junction and bone crest (green) and the mandibular canal (orange). In a model of M1 with the bony socket removed (right) the alveolar bone of the interradicular septum (purple) is superior to the alveolar bone (red) between the incisor (blue) and the apices of the mesial and distal roots (image by B. Johnston).

The effect of time since ovariectomy on the rat mandible is less clear so the studies discussed in this review are subsequently divided into those that are ≤12 weeks in duration and those that are ≥12 weeks in duration after ovariectomy. This time point was chosen since a 12-week period after ovariectomy has been shown to be sufficient to decrease trabecular bone volume at the proximal tibia [35], femoral neck [36], and lumbar vertebrae [37]. Additionally, only studies reporting changes to alveolar bone of the mandible, not maxilla, were included in this review. There is a broader body of literature to support changes to alveolar bone in the mandible of the OVX rat model and by limiting this review to the mandible, with detailed descriptions of the regions of interest (ROI) used in the studies, the changes to alveolar bone are standardized and more focused.  Figure 3 is an image of a hemimandible from a Sprague-Dawley rat with key landmarks and directions highlighted and serves as a guide to the specific study ROI discussed in the next section. Similarly, Figures 4 through 6 are included to assist the reader in identifying regions that have been analyzed in the studies that are discussed in this review.  Figure 4 is a sagittal slice through the first molar (M1) with the interradicular septum containing alveolar bone highlighted.  Figure 5 is a three-dimensional (3D) rendering of the 4 roots of M1 (mesial, lingual, buccal, and distal roots) and shows how these roots enclose the alveolar bone of the interradicular septum.  Figure 6 is a 3D rendering of M1 in its bony socket and viewed as a cut-away to show landmarks used to define alveolar bone ROI.

### 4.1. Short-Term Effects of Ovariectomy on Mandibular Health: Findings from Studies Less Than 12 Weeks after Ovariectomy

Since robust ovariectomy-induced changes to bone structure at the proximal tibia, femur neck, and lumbar vertebrae are certainly manifest by 12 weeks and such changes only begin to be detectable earlier, 2–7 weeks after ovariectomy, it is likely difficult to detect changes in alveolar bone in rats prior to 12 weeks after ovariectomy. Thus, in only 3 of the 6 studies with an ovariectomy duration < 12 weeks, ovariectomy reduced either alveolar bone structure [38, 39] or density [40] (Table 1). In the studies that showed an effect from the ovariectomy, the time since ovariectomy was approximately 9 weeks. Of those studies, rats were ovariectomized at 17, 25, and 26 weeks of age [38–40]. The first study used histomorphometry to measure the M1 sagittal surface containing the central sulcus of the occlusal surface and both the mesial and distal root canals. The ROI was the entire interradicular septum of M1 extending from the furcation roof to the mesial and distal root apices (Figure 4). Relative to the sham group, there was lower bone volume and trabecular number with higher trabecular separation but no difference in trabecular thickness between the sham and OVX groups.

Similar results were reported by a study that also investigated alveolar bone 9 weeks after ovariectomy, the only difference being an older age at ovariectomy, 25 versus 17 weeks [39]. The hemimandibles were scanned via microcomputed tomography (*μ*-CT) between the mesial and distal roots of M1 at a resolution of 50 *μ*m. The ROI was delineated inferiorly by a plane connecting the apices of the buccal and lingual roots (Figure 5) and superiorly by a contour along the interradicular septum (Figure 4). Relative to the sham group, there was lower bone volume and trabecular thickness with higher trabecular separation. There was no difference between the trabecular number of the sham and OVX groups.

Changes in the alveolar bone density of Sprague-Dawley rats ovariectomized at 26 weeks of age and maintained for the same 9 weeks after ovariectomy were also reported [40]. To measure the tissue mineral density (TMD) distribution, hemimandibles were cut into 5 mm sections and scanned via *μ*-CT at a resolution of 20 *μ*m. The ROI was a volume of alveolar bone extending 200 *μ*m from the surface of each tooth along the 5 mm section; if visualized in 3D the ROI would be a 200 *μ*m thick cast of the tooth surfaces in direct contact with the alveolar bone. This ROI included both the periodontal ligament (~150 *μ*m) and the alveolar bone in direct contact with the tooth surface (~50 *μ*m). There was a higher variability in the mineralization of alveolar bone in the OVX group than in the sham group. This higher variability implied more immature bone formation due to accelerated bone remodeling. In summary, these studies have shown that by 9 weeks after ovariectomy there is a reduced alveolar bone volume and increased trabecular separation [38, 39] and the stability of the alveolar bone directly supporting the molars is also compromised [40].

Of the studies with an OVX duration < 12 weeks that did not report an effect on alveolar bone, two had times after ovariectomy of less than 9 weeks [41, 42] and one had a time after ovariectomy of exactly 9 weeks [43]. Methodological differences among the studies with an OVX duration of approximately 9 weeks may also explain why one study reported no effects [43] while 3 others reported effects [38–40]. The study with the shortest study period after ovariectomy measured mandibular BMD in Sprague-Dawley rats ovariectomized at 4 weeks of age and X-ray radiographs of hemimandibles were taken 4 weeks after ovariectomy [41]. The ROI began 1.5 cm mesial to M1 and extended until the end of the alveolar bone supporting the distal root of M3 (Figure 3). The ROI was delineated inferiorly by the crest of the incisor root and superiorly by the contours of the molar roots (Figure 6). This ROI included the alveolar bone supporting M1–M3 and the surrounding cortical bone. There was no difference between the BMD of the sham and OVX groups. Alveolar bone loss was also measured by calculating the difference in height between the cementoenamel junction (CEJ) and the bone crest at the midpoint of the mesial root of each molar (Figure 6). There was no difference in alveolar bone loss between the sham and OVX groups.

At 5 weeks after ovariectomy, the mandibular BMD of Sprague-Dawley rats ovariectomized at 13 weeks was measured using DXA [42]. The ROI of left hemimandibles was a rectangle extending from the angle mesial to M1 until the distal root of M3 (Figure 3). The following were included in the ROI: molars, alveolar bone, cortical bone, and the incisor root. There was no difference in BMD between the sham and OVX groups. Additionally, the mandibular bone area fraction and area moment of inertia were measured using coronal sections (Figure 3) of the distal most aspect of the M1 mesial root (Figure 5). An image of the newly exposed surface constituted the ROI and included the alveolar bone, cortical bone, and incisor root. There was no difference between bone area fraction or area moment of inertia between the sham and OVX groups.

Even 9 weeks after ovariectomy, Wistar rats ovariectomized at 17 weeks reported no difference between the mandibular BMD of the sham and OVX groups [43]. The mandibular BMD was measured by DXA equipped with small animal software. The ROI was a rectangle encompassing the alveolar, condylar, and coronoid processes; the molar crowns and incisor were removed (Figure 3). Since changes in alveolar bone structure [38, 39] and density [40] have been reported by ~9 weeks after ovariectomy, it is possible that DXA may not be sensitive enough to detect the ovariectomy-induced changes in the rat mandible. Studies that follow for a longer time period after ovariectomy are likely needed to detect ovariectomy-induced loss of mandibular BMD by DXA. Future studies that use *μ*-CT to measure changes in alveolar bone structure in response to ovariectomy are also needed. It is likely that *μ*-CT would detect a more subtle change in alveolar bone after a shorter amount of time after ovariectomy than DXA due to its superior resolution, ability to quantify structural changes, and highly specific ROI in three-dimensions (3D). Also, in order to detect a robust change in alveolar bone after ovariectomy, that can be correlated with changes at other typical skeletal sites such as the long bones and lumbar vertebrae, a time after ovariectomy of greater than 12 weeks is likely needed.

### 4.2. Longer-Term Effects of Ovariectomy on Mandibular Health: Findings from Studies That Are 12 Weeks or Longer after Ovariectomy

All of the studies with an ovariectomy duration of longer than 12 weeks (Table 2) report a reduction in bone mineral and/or structural changes in alveolar bone. Of these 9 studies, 5 reported changes in alveolar bone structure [44–48], 3 reported significant reductions in the BMD of alveolar bone [49–51], and a single study reported a decrease in the cortical thickness of alveolar bone [52].

Two studies used conventional histomorphometric methods to report changes in alveolar bone structure following ovariectomy [44, 47]. One study ovariectomized rats at 6 weeks of age and studied them until 12 weeks after ovariectomy [44]. To measure the mandibular histomorphometry, hemimandibles were sectioned coronally into 50 *μ*m thick slices (Figure 3). The ROI was a rectangle, with an area of 0.135 mm^2^, inferior to the apices of the buccal and lingual roots of M1 (Figure 5) and superior to the mandibular canal (Figure 6). There was a lower bone volume in the OVX group than in the sham group. Another study ovariectomized Sprague-Dawley rats at 26 weeks and maintained them for 29 weeks [47]. To measure the mandibular bone area fraction and the area moment of inertia, the left hemimandibles were sectioned coronally (Figure 3) between the mesial and buccal roots of M1 (Figure 5) and also between the roots of M2. The ROI for the bone area fraction was the entire surface of the M1 section with the molar crown/roots and incisor removed. The ROI for the area moment of inertia was the entire surface of the M2 section with the incisor removed. The bone area fraction of the OVX group was lower than the sham group. There was no difference between the moment of inertia between sham and OVX groups.

The remaining 3 studies to report changes in alveolar bone structure used *μ*-CT [45, 46, 48]. To measure the mandibular morphometry of rats ovariectomized at 11 weeks and maintained for 16 weeks, left hemimandibles were scanned via *μ*-CT beginning at the mesial plane of M1 and extending 25 slices toward the distal root (Figure 5); the scan was at a resolution of 15 *μ*m [45]. The ROI was delineated superiorly by the apex of the M1 mesial root (Figure 5) and inferiorly by the crest of the incisor socket (Figure 6). The buccal and lingual walls of cortical bone that flanked the ROI were removed. Relative to the sham group, there was a lower bone volume, lower trabecular thickness, higher trabecular separation, and a higher structure model index in the OVX group. A higher structure model index indicated a shift in trabeculae shape from plate-like to rod-like; rod-like trabeculae are thinner trabeculae and are thus indicative of structurally compromised alveolar bone.

Another study measured the mandibular morphometry of rats ovariectomized at 28 weeks and studied 17 weeks later by scanning the left hemimandibles using *μ*-CT. This analysis was done between the mesial and distal borders of M1 (Figure 5) at a resolution of 16 *μ*m [46]. The ROI was an interpolated shape encompassing the alveolar bone from the apices of the buccal and lingual roots (Figure 5) to the crest of the incisor (Figure 6) and from the mesial to distal surfaces of the M1 interradicular septum (Figure 5). Relative to the sham group, there was a lower trabecular number and connectivity density in the OVX group but no differences in bone volume or trabecular thickness between the sham and OVX groups. A higher resolution *μ*-CT scan and an ROI limited to the interradicular septum (Figure 4) are likely needed to observe changes in bone volume or trabecular thickness.

To investigate longer-term changes in mandibular morphometry, the right hemimandibles of rats ovariectomized at 26 weeks and studied 52 weeks later were scanned using *μ*-CT at a resolution of 20 *μ*m [48]. The ROI was the interradicular septum of M1 delineated inferiorly by a straight line between the mesial and distal roots (Figure 4). Only a single sagittal slice exposing the interradicular septum of M1 was used for the ROI, not the entire volume of the interradicular septum. Relative to the sham group there was a lower bone volume, a lower trabecular thickness, a lower trabecular number, and a higher trabecular separation in the OVX group, indicating compromised structure of alveolar bone.

Of the studies to evaluate changes in mandibular BMD following ovariectomy using DXA, none reported any changes in BMD while each of the studies reported changes in mandibular density or structure by either pQCT [51], *μ*-CT [46], or histomorphometry [47]. Of the 3 studies to evaluate changes in mandibular density by pQCT, all 3 reported a loss of BMD after ovariectomy [49–51]. These studies provide evidence that mandibular BMD measured by DXA cannot represent the changes in alveolar bone following ovariectomy in the rat and that higher resolution techniques such as pQCT or *μ*-CT are needed.

In a study of rats ovariectomized at 35 weeks and studied at 13 weeks after ovariectomy, the BMD of hemimandibles was measured via pQCT between the mesial root of M1 and the distal root of M2 (Figure 3) at a voxel size of 100 *μ*m and a section thickness of 750 *μ*m [49]. The ROI was the entire surface of each coronal section with the molar crowns, roots, and the incisor root removed; this included both trabecular and cortical bone. Relative to the sham group, there was a lower total trabecular and cortical BMD in the OVX group.

Another study in younger ovariectomized rats (13 weeks old) that were studied 16 weeks after ovariectomy scanned hemimandibles using pQCT from the mesial border of M1 to the distal border of M3 (Figure 3) [51]. The ROI was the surface of each coronal pQCT section with the molar crowns/roots and the incisor root removed. The lowest trabecular BMD in the OVX group compared to the sham group was observed 3.5 mm from the mesial border of M1. There was no difference between the sham group and the OVX group cortical BMD. Additionally, the hemimandibles of rats ovariectomized at 26 weeks and maintained for 16 weeks after ovariectomy were scanned via pQCT at one 750 *μ*m thick coronal slice through the midpoint of M1 (Figure 3) at a resolution of 100 *μ*m [50]. The ROI was the entire surface of the coronal slice with the molar crown/roots and the incisor root removed. There was a lower total mandibular BMD in the OVX group compared to the sham group.

A long-term study of rats ovariectomized at 13 weeks and maintained for 52 weeks after ovariectomy measured changes in mandibular cortical bone thickness [52]. Left hemimandibles were exposed to a 70 kV, 7 mA X-ray source and images were digitally captured at a resolution of 12.5 line pairs/mm with a total image size of 640 by 480 pixels. The ROI was a computer-generated contoured shape encompassing the lower mandibular border; it began superiorly where the lower border met the incisor root and extended to the most inferior aspect of the lower border. There was a lower mandibular cortical thickness in the OVX group compared to the sham group.

In summary, the longer the time since ovariectomy, the greater the magnitude of the observed changes in alveolar bone structure. Likewise the age of the rat at ovariectomy determines its skeletal maturity, and thus changes in bone density and structure in a mature rat (>3 months of age) skeleton is more likely to mimic those in a mature adult human skeleton. An immature rat (<3 months of age) skeleton would experience competing skeletal growth after ovariectomy and that may skew any ovariectomy-induced changes. Robust changes to alveolar bone following ovariectomy have consistently been reported in studies that analyze mandible outcomes at or after 12 weeks after ovariectomy. The combination of studying a rat with a mature skeleton (at least 12 weeks of age (~3 months) and ideally closer to 6 months of age at ovariectomy to exclude skeletal growth) and a time after ovariectomy of at least 12 weeks would yield changes to alveolar bone structure and density that are optimal to represent postmenopausal bone loss. Future studies developing interventions to preserve alveolar bone should consider this time frame.

## 5. Effect of Dietary Calcium on Mandibular Health in Ovariectomized Rats

In addition to considering age at ovariectomy and time from ovariectomy, dietary calcium levels are also important to consider. Two studies [44, 48] that used the same level of calcium in the diet (1.15% Ca) but studied the rats for different periods of time after ovariectomy reported different percent changes in alveolar bone volume (24% versus 75%). Thus, the observed differences in alveolar bone volume are due to differences in the time after ovariectomy. In other studies that were of similar length after ovariectomy (16 weeks [50] versus 13 weeks [49]), different dietary calcium levels affected mandibular outcomes. For example, a tenfold difference in dietary calcium, of which both levels were considered “low calcium” (0.1% calcium and 0.01% calcium), resulted in a 2.88% and 7.35% decrease in total mandibular BMD, respectively. Thus, mandibular health is dependent on the age at ovariectomy, time after ovariectomy, and the level of dietary calcium. To control for such variation, future studies could use semipurified diet such as the AIN93M that is specially formulated to meet the nutritional needs of the adult rat [53] and facilitates a standardization of the effects of diet on bone outcomes. Although not specifically studied in the ovariectomized rat model, other aspects of a diet including macronutrient or micronutrient content can also likely affect the outcomes of mandibular health if not tightly controlled among studies.

## 6. Estrogen Replacement Therapy Preserves Mandibular Health in Ovariectomized Rats

A single human trial has reported higher alveolar bone density with ERT [24], yet with the ovariectomized rat model it is clear that estrogen treatment can preserve both the periodontium [54, 55] and alveolar bone density [56, 57] and also reduce mandibular bone turnover [58] (Table 3). Rats that were ovariectomized at an unreported age and maintained for 2 weeks were divided into sham, OVX, and OVX + estrogen and were implanted with mini osmotic pumps to continuously deliver either vehicle (sham and OVX groups) or estrogen (OVX + estrogen group) [54]. The treatment that the OVX + estrogen group received was 1.5 *μ*g/day of 17 *β*-estradiol; this dose was not standardized to body weight so it is difficult to place in the context of the other studies. To measure mandibular osteoclastogenesis, right hemimandibles were sectioned into 5 *μ*m thick slices at the M1 mesial root (Figure 5) and stained for tartrate-resistant acid phosphatase (TRAP) activity. The ROI was the buccal periodontium surrounding the mesial root of M1. At 2 weeks after ovariectomy, there was less nuclei/osteoclast observed in the OVX + estrogen group compared to the OVX group. There was no difference in the number of nuclei/osteoclast between the sham and OVX + estrogen groups. Thus, estrogen treatment attenuated the ovariectomy-induced osteoclastogenesis observed in the rat buccal periodontium of M1.

Another study indicates that rats that were ovariectomized at 13 weeks of age were administered 17 *β*-estradiol by injection at a dose of 10 *μ*g/kg for 5 days/week, for 7 weeks after ovariectomy [55]. To measure the periodontal ligament space, left hemimandibles were cut sagittally at the buccal/lingual midpoint (Figure 5) to expose M1–M3 and were scanned via scanning electron microscopy. The ROI was the distance between a molar root surface and the supporting alveolar bone at 3 randomly chosen sites per rat. There was a greater periodontal ligament space in the OVX group than in the sham group. Estrogen treatment inhibited the expansion of the periodontal ligament space and, by extension, alveolar bone resorption.

Rats ovariectomized at 11 weeks received a daily subcutaneous injection of 17 *β*-estradiol at a dose of 20 *μ*g/kg for 11 weeks [56]. To measure the alveolar bone density, hemimandibles were sectioned into 6 *μ*m thick slices in the coronal plane (Figure 3) between the mesial and distal roots of M1 (Figure 5). The ROI was the volume of alveolar bone within 1000 *μ*m of the furcation roof on 5 equally spaced slices within the M1 interradicular septum. There was a lower alveolar bone density in the OVX group compared to the sham group. There was no difference in alveolar bone density between the sham and the OVX + estrogen groups.

A similar study also used Wistar rats ovariectomized at 11 weeks but treated the rats with an oral dose of estriol at 100 *μ*g/kg, 5 days/week for 12 weeks after ovariectomy [57]. To measure changes in trabecular BMD and bone mineral content (BMC), the hemimandibles were scanned using pQCT at a resolution of 100 *μ*m at 11 slices beginning 0.5 mm from the mesial boarder of M1 to the distal border of M3 (Figure 3). The ROI extended from the superior edge of the incisor root (Figure 6) to the molar furcation roof and excluded the molar crown, roots, and surrounding cortical bone. The OVX + estrogen treated group had a higher trabecular BMD and BMC at multiple slices compared to the OVX group; the sites of greatest alveolar bone preservation were the slices directly beneath M1 and M2.

To measure longer-term changes in mandibular bone remodeling rats were ovariectomized at 13 weeks and left untreated for 52 weeks after ovariectomy [58]. After the 52-week period, one group received estrogen treatment for 10 weeks as a subcutaneous injection of 17 *β*-estradiol for 4 days each week at a dose of 10 *μ*g/kg. Fluorochrome bone markers were also administered at 17 and 7 days prior to necropsy. To measure the mandibular histomorphometry, right hemimandibles were sectioned coronally at M2 (Figure 3) into 30 *μ*m thick slices and visualized with a fluorescence microscope to quantify bone turnover. There were two ROIs, the periosteal bone surface around the outside of the mandibular cortical bone and the endosteal bone surface around the trabeculae within the M2 supporting alveolar bone. On the periosteal surface, the mineralizing surface of the OVX group was higher than the OVX + estrogen group. On the endosteal surface, the double-labeled surfaces, mineralizing surfaces, and mineral apposition rates were all higher in the OVX group compared to the OVX + estrogen group. By reducing bone turnover in the rat mandible, estrogen treatment may work to preserve existing alveolar bone mass.

Estrogen treatment reduced osteoclastogenesis, stabilized bone turnover, and therefore preserved alveolar bone mass in the ovariectomized rat. However, the preservation of alveolar bone structure following estrogen treatment remains unclear. Future studies should correlate the preservation of alveolar bone structure following ERT with other key sites rich in trabecular bone that are known to respond to ERT in order to place the bone-sparing effects of ERT on alveolar bone in the context of systemic bone health.

## 7. Conclusions

Our review of the literature indicates that the ovariectomized rat experiences a deterioration of alveolar bone that resembles the loss that can be experienced by postmenopausal women. Moreover, the well-characterized loss of bone mineral and structure that occurs in the long bones and lumbar spine occurs concurrently with a loss of tooth-supporting alveolar bone in the mandible of the ovariectomized rat. This link between the traditional sites of bone loss (hip and lumbar spine) and alveolar bone emphasizes the ability of future intervention studies to have a bimodal effect on skeletal health, targeting both fracture prevention and tooth retention. Some considerations to maximize the benefits of this model include the measurement techniques used, the age at ovariectomy, and the duration for which a rat is studied after ovariectomy. Diet should also be controlled by adopting standardized diets such as AIN93M to ensure that differences among studies are not due to differences in specific nutrients such as calcium.

Ovariectomy-induced changes to alveolar bone in the preclinical rat model of postmenopausal osteoporosis are detected by traditional histomorphometry, pQCT, and *μ*-CT, but not DXA. The rat should be at least 3 months of age when ovariectomized (ideally closer to 6 months of age) and the time after ovariectomy should also be at least 3 months. The alveolar bone region of interest should be limited to the interradicular septum of the first molar because it is the most well-characterized site and appears to respond positively to the established bone sparing effect of estrogens. The capacity of the alveolar bone to respond positively to estrogen replacement therapy highlights the possibility of additional interventions that target bone anabolism and reduce bone turnover. To date alveolar bone turnover in the ovariectomized rat has been reduced by bisphosphonate (alendronate [56] and risedronate [58]) treatment, and alveolar bone formation has been stimulated by calcitonin [58] and intermittent parathyroid hormone treatment [59]. Such studies suggest that alveolar bone may be much more sensitive to strategies targeting systemic bone preservation in the preclinical model of postmenopausal bone loss than previously thought. Future studies investigating bone-preserving strategies for the typical sites of ovariectomy-induced bone loss such as the proximal tibia metaphysis, distal femur epiphysis, femoral neck, and lumbar vertebral bodies should include the alveolar bone of the M1 interradicular septum as a region of interest. Studies that show preservation of alveolar bone as well as skeletal sites that are well-established for bone loss after ovariectomy in this preclinical model will provide an important basis for interventions in postmenopausal women. The tight link between alveolar bone preservation, tooth retention, better nutritional status, and the reduced risk of developing chronic disease begins with the mitigation of postmenopausal bone loss. The ovariectomized rat model has the potential to be a preclinical model of postmenopausal alveolar bone loss and could facilitate future drug and nutritional strategies aimed at tooth retention and thus a reduced risk of developing chronic disease.

## Figures and Tables

**Table 1:** Summary of studies investigating changes in mandibular health in rats less than 12 weeks after ovariectomy.

Rat strain	Sample size per group	Age at OVX (wks)	Time after OVX (wks)	Diet	Technology	Main findings for mandible, compared to sham control	Reference
Sprague-Dawley	*n* = 5	4	4	0.89% Ca	Radiographs	(i) No change in BMD (ii) No alveolar bone loss	[41]

Sprague-Dawley	*n* = 8	13	5	1.00% Ca	DXA, histomorphometry stereology	(i) No change in BMD, bone area fraction, or area moment of inertia	[42]

Fischer	*n* = 8	17	9	Unknown	Histomorphometry	(i) 25% decrease in BV/TV (ii) 17% decrease in Tb.N (iii) 32% increase in Tb.Sp (iv) No change in Tb.Th	[38]

Sprague-Dawley	*n* = 10	26	9	Unknown	*µ*-CT	(i) 7% increase in the tissue mineral density distribution grey values at the 5th percentile (ii) 25% increase in the tissue mineral density distribution coefficient of variation	[40]

Wistar	*n* = 6	17	9	Unknown	DXA	(i) No change in BMD	[43]

Wistar	*n* = 6	25	9	1.17% Ca, 0.91% P	*µ*-CT	(i) 15% decrease in BV/TV (ii) 14% decrease in Tb.Th (iii) 22% increase in Tb.Sp (iv) No change in Tb.N	[39]

BMD, bone mineral density; BV/TV, bone volume; Tb.N, trabecular number; Tb.Sp, trabecular separation; Tb.Th, trabecular thickness; DXA, dual-energy X-ray absorptiometry; *µ*-CT, microcomputed tomography.

**Table 2:** Summary of studies investigating mandibular health in rats for 12 weeks or more after ovariectomy.

Rat strain	Sample size per group	Age at OVX (wks)	OVX duration (wks)	Diet	Technology	Main findings for mandible, compared to sham control	Reference
Wistar	*n* = 5	6	12	1.15% Ca, 0.35% P	Histomorphometry	(i) 24% decrease in BV	[44]

Wistar	*n* = 15	35	13	0.01% Ca	pQCT	(i) 7% decrease in total BMD (ii) 11% decrease in Tb.BMD (iii) 1% decrease in Ct.BMD	[49]

Sprague-Dawley	*n* = 6	13	16	Unknown	DXA, pQCT	(i) No change in BMD (ii) 13% decrease in Tb.BMD (iii) No change in Ct.BMD	[51]

Lewis-Brown-Norway	*n* = 12	11	16	Unknown	*µ*-CT	(i) 18% decrease in BV/TV (ii) 28% decrease in Tb.Th (iii) 67% increase in Tb.Sp (iv) 22% increase in SMI	[45]

Wistar	*n* = 12	26	16	0.1% Ca	pQCT	(i) 3% decrease in total BMD	[50]

Sprague-Dawley	*n* = 11	28	17	1.1% Ca, 0.80% P	DXA, *µ*-CT	(i) No change in BMD (ii) 6% decrease in Tb.N (iii) 19% decrease in Conn.D (iv) No change in BV/TV (v) No change in Tb.Th	[46]

Sprague-Dawley	*n* = 15	26	29	1.0% Ca	DXA, histomorphometry	(i) No change in total or molar region BMD (ii) 8% decrease in bone area fraction (iii) No change in area moment of inertia	[47]

Lewis-Brown-Norway	*n* = 6	13	52	Unknown	Radiograph	(i) 16% decrease in Ct.Th	[52]

Fischer	*n* = 6	26	52	1.15% Ca, 0.88% P, 0.80 IU/g vit. D^3^	*µ*-CT	(i) 75% decrease in BV/TV (ii) 46% decrease in Tb.Th (iii) 58% decrease in Tb.N (iv) 354% increase in Tb.Sp	[48]

BMD, bone mineral density; Tb.BMD, trabecular BMD; Ct.BMD, cortical BMD; BV, bone volume (2D); BV/TV, bone volume (3D); Tb.N, trabecular number; Tb.Sp, trabecular separation; Tb.Th, trabecular thickness; Ct.Th, cortical thickness; SMI, structure model index; Conn.D, connective density; DXA, dual-energy X-ray absorptiometry; *µ*-CT, microcomputed tomography.

**Table 3:** Summary of studies investigating estrogen replacement therapy and mandibular health in ovariectomized rats.

Rat strain	Sample size per group	Age at OVX (wks)	OVX duration (wks)	Estrogen dose; duration (wks)	Main findings for mandible, compared to OVX	Reference
Wistar	*n* = 4	Unknown	2	17*β*-estradiol 1.5 *μ*g/day continuous infusion; 2	(i) Less nuclei (osteoclasts)	[54]

Sprague-Dawley	*n* = 5	13	7	17*β*-estradiol 10 *μ*g/kg 5 days/wk; 7	(i) 36% less periodontal ligament space	[55]

Wistar	*n* = 14	11	11	17*β*-estradiol 20 *μ*g/kg daily; 11	(i) 41% greater bone density	[56]

Wistar	*n* = 15	26	16	Estriol 100 *μ*g/kg 5 days/wk; 12	(i) Improved trabecular BMD and BMC	[57]

Sprague-Dawley	*n* = 9	13	62	17*β*-estradiol 10 *μ*g/kg 4 days/wk; 10	(i) 38% less periosteal mineralizing surface (ii) 88% less endosteal double-labeled surface (iii) 51% less endosteal mineralizing surface (iv) 71% less endosteal mineral apposition rate	[58]

OVX, ovariectomized; BMD, bone mineral density; BMC, bone mineral content.
